# Conceptual framework for data harmonisation in mental health using the International Classification of Functioning, Disability and Health: an example with the R2D2-MH consortium

**DOI:** 10.1136/bmjment-2024-301283

**Published:** 2024-11-28

**Authors:** Melissa H Black, Jan Buitelaar, Tony Charman, Christine Ecker, Louise Gallagher, Kristien Hens, Emily Jones, Declan Murphy, Yair Sadaka, Marie Schaer, Beate St Pourcain, Dieter Wolke, Stef Bonnot-Briey, Thomas Bourgeron, Sven Bölte

**Affiliations:** 1 Center of Neurodevelopmental Disorders (KIND), Department of Women’s and Children’s Health, Centre for Psychiatry Research, Karolinska Institute, Stockholm, Sweden; 2 Curtin School of Allied Health, Curtin University, Perth, Western Australia, Australia; 3 Donders Institute for Brain, Cognition and Behaviour, Radboud University, Nijmegen, The Netherlands; 4 Department of Cognitive Neuroscience, Radboud Universiteit, Nijmegen, The Netherlands; 5 Karakter Child and Adolescent Psychiatric University Centre, Nijmegen, the Netherlands; 6 Department of Psychology, Institute of Psychiatry, Psychology & Neuroscience, King's College London, London, UK; 7 Department of Child and Adolescent Psychiatry, Psychosomatics and Psychotherapy, Autism Research and Intervention Center of Excellence, University Hospital Frankfurt, Goethe University Frankfurt, Frankfurt am Main, Germany; 8 Institute for Translational Neurodevelopment, King's College London, London, UK; 9 Child and Youth Mental Health Collaborative, SickKids, Centre for Addiction and Mental Health and University of Toronto, Toronto, Ontario, Canada; 10 Department of Psychiatry, School of Medicine, Trinity College, Dublin, Ireland; 11 Department of Philosophy, University of Antwerp, Antwerpen, Belgium; 12 Centre for Brain and Cognitive Development, University of London, London, UK; 13 Department of Forensic and Neurodevelopmental Sciences, Institute of Psychiatry, Psychology, and Neuroscience, King's College London, London, UK; 14 Neurodevelopmental Research Center, Mental Health Institute, Be’er Sheva, Israel; 15 KI Research Institute, Kfar Malal, Israel; 16 Faculty of Health Sciences, Ben-Gurion University of the Negev, Be’er-Sheva, Israel; 17 Autism Brain & Behavior Lab, Department of Psychiatry, Faculty of Medicine, University of Geneva, Geneva, Switzerland; 18 Max Planck Institute for Psycholinguistics, Nijmegen, The Netherlands; 19 MRC Integrative Epidemiology Unit, University of Bristol, Bristol, UK; 20 Division of Health Sciences, Warwick Medical School, University of Warwick, Coventry, UK; 21 Department of Psychology, University of Warwick, Coventry, UK; 22 HANDI-VOICE, Paris, France; 23 PAARI et fédération AUTOP-H, Paris, France; 24 Autism-Europe, Brussels, Belgium; 25 Human Genetics and Cognitive Functions, Institut Pasteur, UMR3571 CNRS, IUF, Université Paris Cité, Paris, France; 26 Child and Adolescent Psychiatry, Stockholm Health Care Services, Stockholm, Sweden; 27 Curtin Autism Research Group, Curtin School of Allied Health, Curtin University, Perth, Western Australia, Australia

**Keywords:** PSYCHIATRY

## Abstract

**ABSTRACT:**

**Introduction:**

Advancing research and support for neurologically diverse populations requires novel data harmonisation methods that are capable of aligning with contemporary approaches to understanding health and disability.

**Objectives:**

We present the International Classification of Functioning, Disability and Health (ICF) as a conceptual framework to support harmonisation of mental health data and present a proof of principle within the Risk and Resilience in Developmental Diversity and Mental Health (R2D2-MH) consortium.

**Method:**

138 measures from various mental health datasets were linked to the ICF following the WHO’s established linking rules.

**Findings:**

Findings support the notion that the ICF can assist in the harmonisation of mental health data. The high level of shared ICF codes provides indications of where items may be readily harmonised to develop datasets that may align more readily with contemporary approaches to understanding health and disability. Although the linking process necessarily entails an element of subjectivity, the application of established rules can increase rigour and transparency of the harmonisation process.

**Conclusions:**

We present the first steps towards data harmonisation in mental health that is compatible with contemporary approaches in psychiatry, being more capable of capturing diversity and aligning with more transdiagnostic and neurodiversity-affirmative ways of understanding data.

**Clinical implications:**

Our findings show promise, but future work is needed to address quantitative harmonisation. Similarly, issues related to the traditionally ‘pathophysiological’ frameworks that existing datasets are often embedded in can hinder the full potential of harmonisation based on the ICF.

**WHAT IS ALREADY KNOWN ON THIS TOPIC**

- Retrospectively harmonising existing data collections into larger scale datasets is increasingly of interest in mental health. New methods are required to align with contemporary paradigms in psychiatry.

**WHAT THIS STUDY ADDS**

- We present the International Classification of Functioning, Disability and Health (ICF) as a conceptual framework to support harmonisation efforts in mental health and present a proof of principle within the Risk and Resilience in Developmental Diversity and Mental Health (R2D2-MH) project and consortium.

**HOW THIS STUDY MIGHT AFFECT RESEARCH, PRACTICE OR POLICY**

- We present the first steps of how the ICF may be used as a conceptual framework for data harmonisation in mental health which may more readily align with contemporary paradigms in mental health. This work may pave the way for new data harmonisation and research that aims to support neurologically diverse individuals.

## Introduction

 Retrospectively harmonising existing data collections into larger scale datasets is increasingly of interest in psychiatric research. Such methods can maximise research efforts and funding, create larger sample sizes and enable existing data to be re-examined in new ways, maximising their research potential.^1 2^ Despite the many benefits of data harmonisation to psychiatric research, this process can be challenging. Retrospective data harmonisation involves aligning data collected from different studies and measures with the aim of improving comparability across similar measures.^1 2^ Here, limitations to comparability, foremost variability in measures and populations within individual datasets, driven by the absence of standardised commonly agreed-upon measures across studies and cohorts, can pose challenges to harmonisation.^1 2^ These challenges may be particularly pronounced in psychiatry, which is characterised by diverse biopsychosocial data. Nevertheless, aggregating such data is necessary to psychiatry where there is considerable between-person and within-person variation in biological, cognitive, psychological and socioenvironmental characteristics that must be captured.^3^ Even when challenges to harmonisation are overcome, current methods may result in harmonised datasets that are not optimally suited to advancing psychiatric research, often remaining embedded in paradigms that more contemporary approaches have superseded.

### Three paradigms for contemporary data harmonisation

We propose that for harmonised data to be truly beneficial to psychiatric research, the methods through which data are harmonised must result in datasets that align with contemporary paradigms in the field, namely transdiagnostic, neurodiversity and positive psychology approaches.

First, harmonisation methods must be capable of facilitating transdiagnostic and continuous approaches. Transdiagnostic approaches recognise that there can be significant interindividual variability and overlap between underlying mechanisms and presentations, even within supposedly discrete diagnostic categories.^4 5^ Rather than attempting to confine characteristics within specific diagnostic labels, transdiagnostic approaches conceptualise characteristics as lying within a multidimensional space where particular features may be shared across diagnoses. Such approaches are well established in psychology and are increasingly favoured in psychiatry, with initiatives such as the National Institute of Mental Health Research Domain Criteria Initiative (RDoC) encouraging researchers to adopt perspectives that look beyond diagnostic categories.^6^ Yet, individual datasets generated by preclinical studies, clinical trials and other initiatives are frequently developed within common diagnostic frameworks. Thus, although transdiagnostic approaches are preferred, harmonised datasets may remain embedded in more categorical framings, often failing to capture the nuance and heterogeneity within and between populations.

At the same time, there is a more recent shift towards recognising heterogeneity as part of natural human development.^4^ The neurodiversity paradigm, most commonly applied to neurodevelopment, but is also seen in mental health,^7^ argues that neurological diversity is not pathological but part of natural biological human variation. Further, while more pathogenic models more strongly emphasise biological mechanisms, neurodiversity emphasises the fit between an individual and their surrounding context. Neurodiversity-affirmative data (data that align with core tenants of the neurodiversity paradigm) could extend transdiagnostic approaches, whereby profiles are examined across multiple systems levels (biological to environmental and their interaction).

Third, harmonised data in psychiatry should provide opportunities to expand beyond pathogenic risk and deficit paradigms to those encompassing positive outcomes such as well-being (ie, feeling good and having positive functioning).^8^,^10^ Salutogenesis, positive psychology and resilience are three different but highly compatible approaches that may benefit psychiatry when applied to big data. Though having different theoretical bases, these approaches broadly advocate for a shift from pathology and risk towards promoting positive health and functioning. Although these paradigms are not new, they have received limited attention in psychiatry. Such approaches present opportunities to shift focus to promoting positive health and functioning rather than solely on eradicating risks or deficits.^11^ Importantly, under these approaches, all individuals can experience positive outcomes. At a more fundamental level, it is also increasingly recognised that examining strengths and positive outcome alongside deficit and risk is required for person-centred support and a more holistic understanding of neurodivergence.^12^

### A proposal: the International Classification of Functioning, Disability and Health as a conceptual framework for data harmonisation

**Figure 1:**
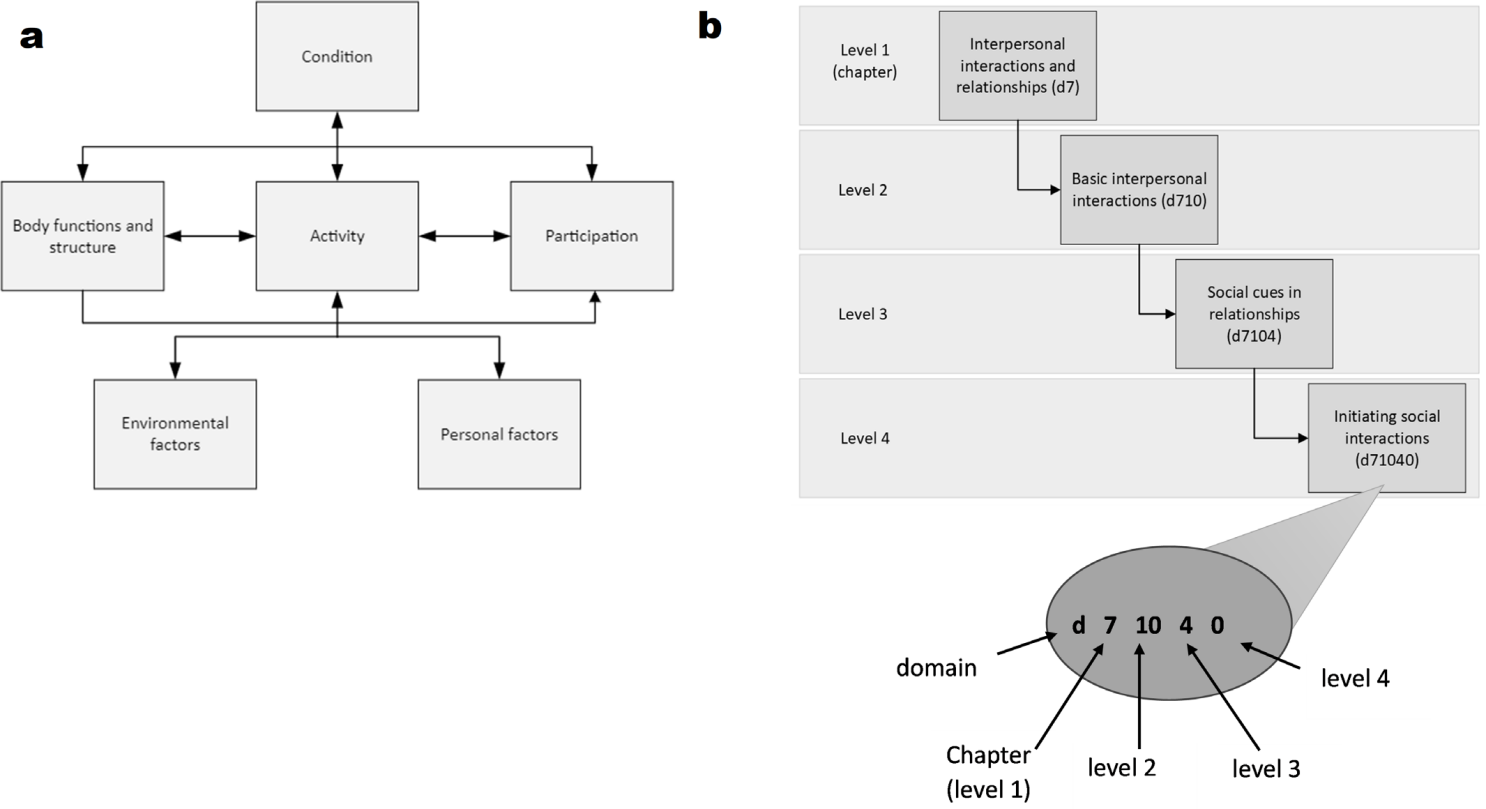
(**a**) International Classification of Functioning, Disability and Health (ICF) framework. (**b**) Example of the hierarchical organisation of the ICF. At the highest level are domains (body functions, body structures, activity and participation, environmental and personal factors) within which sit chapters (level 1). Within chapters are up to three levels of information (levels 2–4) providing increasingly detailed information. Codes refer to how information is coded within the ICF. Codes comprise a prefix corresponding to a particular domain (b: body functions, s: body structures, d: activities and participation, e: environmental factors, i: personal factors), followed by up to four numbers that correspond with the chapter (level 1) and second, third and fourth levels of detail. In the figure example, it can be seen that codes refer to the activity and participation domain (d), chapter 7 (d7), followed by increasingly detailed information, with level 4 being the most detailed.

We propose that the World Health Organization (WHO) International Classification of Functioning, Disability and Health (ICF)^13^ provides a conceptual framework for harmonising diverse and large-scale datasets in a way that aligns with contemporary paradigms needed to transform research and support in neurological diversity. The ICF focuses on functioning, conceptualised as the result of the interaction between an individual, their health condition and their environment (figure 1a,b).^13^ In this way, the ICF accounts for the impact of an individual’s environment, at micro, meso and macro levels, on functioning. It comprises two parts; ‘Functioning and Disability’ composed of body functions (physiological functions, including psychological functions), body structures (anatomical body parts) and activities and participation (execution of tasks or involvement in life situations). The second part, ‘Contextual Factors’, comprises environmental factors (factors outside of an individual including attitudinal, physical and societal) and personal factors (factors inherent to a person, such as age, gender, socioeconomic status, ethnicity and cultural background that can influence functioning).^13^

**Figure 2:**
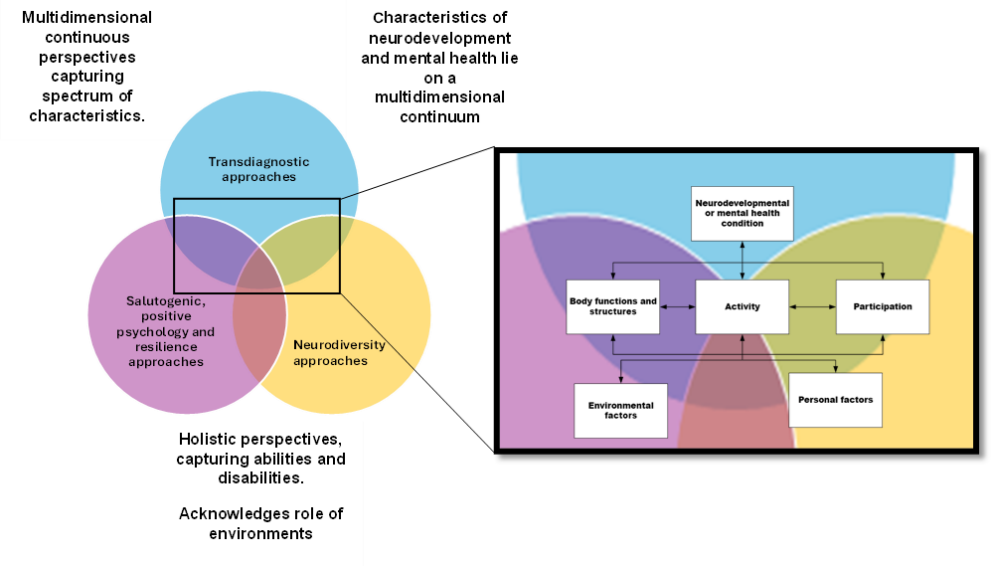
Pictorial representation of the ICF’s alignment with three paradigm shifts that big data in psychiatry must be compatible with. ICF, International Classification of Functioning, Disability and Health.

Through its view of functioning, the ICF aligns with several contemporary approaches in psychiatry (figure 2). First, functioning is not concerned with diagnostic status. It applies to the entire population, where domains of functioning are relevant to all individuals who show various degrees of functioning across domains.^13^ At the same time, because functioning is viewed on a spectrum ranging from ability to disability, it can capture internal (eg, body functions, body structures) and external (activities and participation, environmental) strengths and resources, providing the basis for supporting approaches that promote well-being and positive outcomes. The ICF’s biopsychosocial framework also effectively reconciles biomedical and social models of disability providing opportunities for more holistic and harmonious understandings across disciplines and stakeholder groups.^14^ As it aligns with paradigms more readily acceptable by community stakeholders (eg, populations of interest and their families) while simultaneously integrating biomedical perspectives, work based on the ICF may assist in reconciling the sometimes disparate views between scientists and communities.^14^

From a more practical perspective, the ICF comprises a comprehensive classification system,^15 16^ making it readily available to facilitate the translation of diverse and multimodal clinical and phenotypic data to ICF vocabulary. The classification system comprises almost 1700 hierarchically organised codes (eg, figure 1b for code examples and explanation of coding structure) for classifying body functions (k=531 codes), body structures (k=329 codes), activities and participation (k=552 codes) and environmental factors (k=273 codes).^17^ Though the ICF does not provide a classification system for personal factors, Grotkamp *et al*^18^ have developed an ICF-aligning personal factor classification system that can supplement the ICF (k=79 codes). This classification system presented by the ICF is universally recognised and provides a common language for describing functioning, disability and health across disciplines and projects, enhancing data sharing and collaborative practices.^13^ The WHO and the ICF Research Branch provide clear and systematic guidelines for linking health information to the ICF classification system,^15 16^ and there is an extensive body of work that has applied the ICF across diverse health conditions and life stages, demonstrating its wide and global application. As such, the ICF is a readily available framework and classification system through which to enhance the transparency and reproducibility of the harmonisation process.

### Risk and Resilience in Developmental Diversity and Mental Health (R2D2-MH) in a nutshell

**Figure 3:**
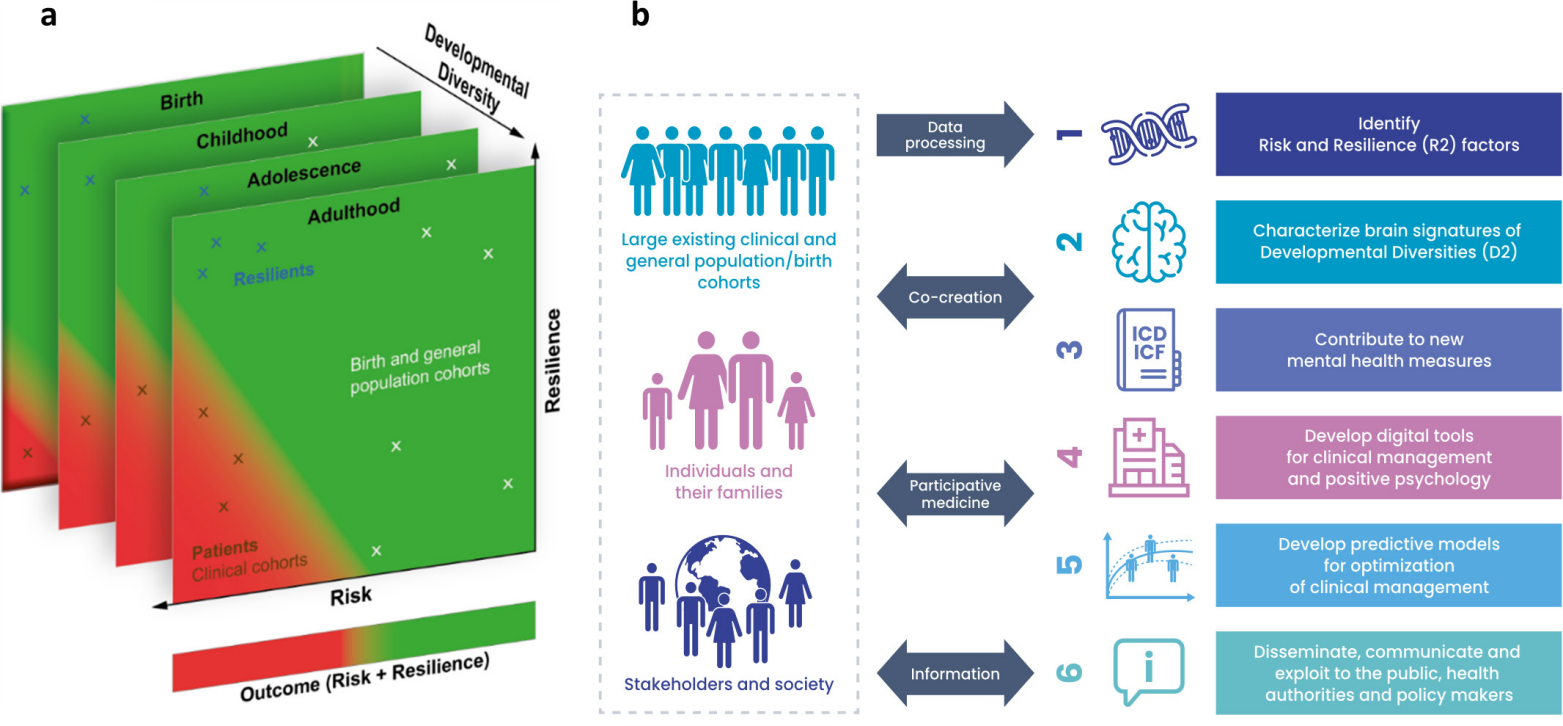
(**a**) Risk and Resilience in Developmental Diversity and Mental Health (R2D2-MH) investigates large-scale and deep-phenotyped cohorts to identify individuals at similar risks but with different mental health outcomes. Clinical cohorts are biased towards participants with poor outcomes. Resilient individuals—defined as exposed to high risk but with low levels of symptoms and good outcome—are detected in preterm cohorts, in the general populations and in relatives of individuals with neurodevelopmental conditions. (**b**) Overview of the R2D2-MH project.

Risk and Resilience in Developmental Diversity and Mental Health (R2D2-MH; https://www.r2d2-mh.eu/) is a 5-year project co-funded by the European Commission (EU contribution € 7,85 million) that started in September 2022 which will attempt to use this approach and will provide the basis for this proof of concept. It includes 17 Partners and 10 Associated Partners across Europe, Israel, Australia and Canada. R2D2-MH proposes a double paradigm shift to improve the well-being of neurologically diverse people and their families. It moves (1) from risk-focused studies towards understanding and promoting resilience, and (2) from a diagnosis-based approach to a developmental diversity approach that captures well-being and functioning across the human lifespan (figure 3a). R2D2-MH investigates two highly prevalent early risks for negative mental health outcomes at multiple levels: prematurity and neurodevelopmental conditions. The project has four main ambitions: (1) to provide the largest European multiscale dataset on early human brain development and mental health outcomes; (2) to identify biological mechanisms of resilience to the adverse effects of neurodevelopmental conditions; (3) to evaluate existing tools and codevelop new tools and indicators; and (4) to establish predictive models to guide personalised interventions (figure 3b). For this project, there is a pressing need for data harmonisation methods capable of reimagining existing data drawn from diverse models of thinking to more continuous and transdiagnostic conceptualisations that can better capture nuance and heterogeneity. It is envisioned that the ICF will provide such a harmonisation method required for R2D2-MH to fulfil its potential.

### Current objectives

Our objectives for this study are to: (1) introduce the R2D2-MH consortium, (2) provide an overview establishing the need for novel approaches for harmonisation in psychiatry, (3) introduce the ICF as a potential conceptual framework for harmonisation and (4) demonstrate this process through a proof of principle linking multimodal and diverse datasets to the ICF. This proposal does not present quantitative harmonisation (ie, how this mapping translates into quantification) but discusses future avenues in this area.

## Methods

### Data sources

The ICF was used as a conceptual framework for the harmonisation of data across cohorts. To identify eligible datasets for the application of the ICF, work package leaders in R2D2-MH first compiled lists of candidate datasets, detailing the purposes, samples, availability of data and variables of interest. Here we use an initial selection of a subset of datasets selected for inclusion in R2D2-MH (summarised in online supplemental table 1). Additional measures planned to be conducted as part of R2D2-MH were also identified by the work package leaders for inclusion. Included datasets were the EU-AIMS Longitudinal European Autism Project (LEAP),^19^ the Preschool Brain Imaging and Behaviour Project (PIP) (https://www.aims-2-trials.eu/pip/), Evaluation of MRI to Predict Neurodevelopmental Impairment in Preterm Infants (ePRIME),^20^ the SOSTA-net Randomized Controlled Trial (RCT),^21^ the ASD-specific Frankfurt Early Intervention Programme for ASD RCT (A-FFIP),^22^ the Neurobiology and Treatment of Adolescent Female Conduct Disorder (FemNAT-CD)^23^ and the developing Human Connectome Project (dHCP).^24^ The datasets cover a broad range of cohorts in neurodevelopmental conditions and mental health, including diagnoses of autism, attention-deficit hyperactivity disorder, intellectual disability, developmental delay, epilepsy and conduct disorders, with ages ranging from infants born preterm to adults. Most of the datasets also included neurotypical comparative samples. A description of the data sources is included in online supplemental table 1 and all measures are contained in online supplemental table 2.

### Linking procedure

Linking of measures to the ICF was conducted in accordance with ICF linking guidelines established by the WHO and ICF Research Branch.^15 16^ For questionnaires, identified measures were located, and individual items were extracted.^15^ Each item was then reviewed within the broader context and purpose of the measure to identify the concept most relevant to be linked (main concept), as well as additional concepts containing other relevant information. For performance measures (ie, cognitive tasks), the aim of the measurement was identified as the main concept.^16^ For example, the item ‘good attention span’ would result in a main concept of ‘attention span’. Further examples of the extracted main and additional concepts are displayed in online supplemental tables 3 and 4. As recommended by the most recent linking guidelines, the perspectives and response options were also extracted from the measures. Perspectives refer to the underlying purpose of the measure and were categorised according to definitions provided by Cieza *et al*.^15^ The extracted main and additional concepts were subsequently linked to the ICF by applying established ICF linking rules and decision-making processes.^15^ Here, we used the ICF–Child and Youth version, as this version represents the most comprehensive version of the ICF, containing codes also relevant to both adults and developing individuals.^17^ Personal factors (ie, age, sex/gender, cultural background) are ordinarily not coded in the ICF, but to capture this information, concepts identified as personal factors were linked to the personal factor classification system developed by Grotkamp *et al*.^18^ The workflow for the linking process is displayed in online supplemental figure 1. Given that datasets drawn on for harmonisation purposes may not always contain item-level data, scale-level data were also extracted and linked to the ICF following the same process described for item-level data. This linking is presented in 25. Linking was completed by one researcher. To enhance the rigour of the process, linking for a subset of measures (1599 items in total) was compared with linking performed by two other linkers. Inter-rater agreement was then calculated for the 1599 items, where codes assigned to each item were examined at the second level and assigned a binary classification of 1 (agreement) or 0 (no agreement). Calculations demonstrated substantial agreement (k=0.75, CIs: k*=*0.73 to 0.77) in codes assigned between the linkers. Following the calculation of the inter-rater agreement, areas of discrepancy were observed and resolved, with linking for the remaining measures and items then refined and finalised. All linking was conducted by researchers who had previous experience with the ICF and ICF linking. To enhance transparency and to enable researchers to use the linking, we provide all linking at the item and scale level in 25. For the purposes of results presentation, linking is reported as absolute and relative frequencies at the domain, chapter and second level of the ICF.

## Results

### Mapping measures collected in R2D2-MH cohorts to the ICF

**Figure 4:**
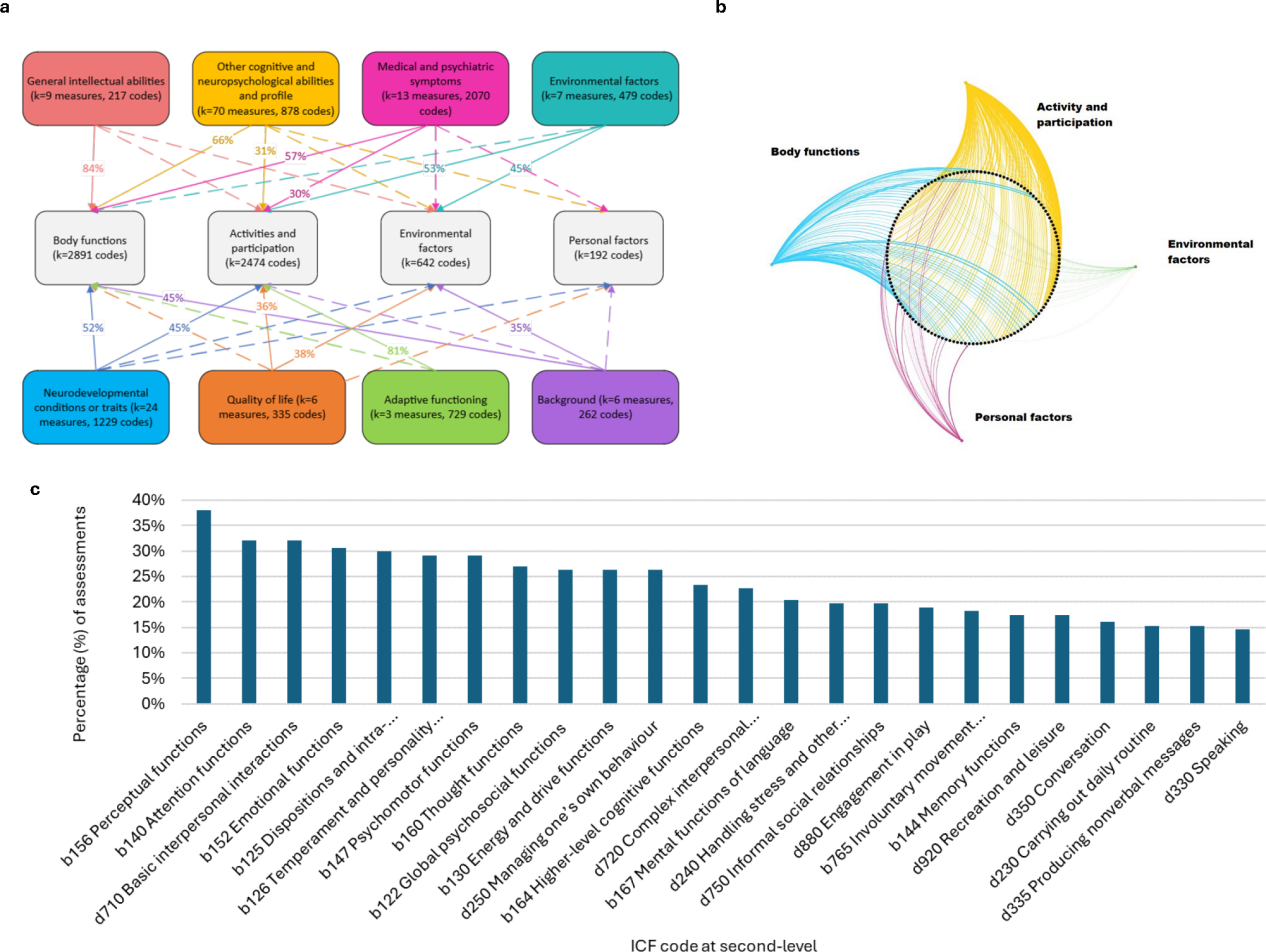
(**a**) Results of the ICF mapping process of measures to the ICF domains. Dotted lines indicate where the frequency of codes was <20%. No codes were applied to body structures, so this domain was not included. For example, general intellectual abilities include nine measures linked to 217 ICF codes, of which 84% related to the body function domain of the ICF. (**b**) Distribution of codes at ICF domain level for individual measures. Black dots represent individual measures, and lines represent links to each domain. An interactive version of this figure displaying all measures and their connections to individual domains can be accessed here: https://melissahblack.github.io/R2D2-MH-Content-Harmonization-using-the-ICF/. Visualisations developed using Gephi ^30^ and the sigma.js plugin developed the InteractiveVis project at the Oxford Institute.^31^ Individual linking for all measures are accessible in ^25^ (**c**) Second-level ICF codes most frequently linked to measures. Only codes linked to 15% or more of the measures are displayed. ICF, International Classification of Functioning, Disability and Health.

A total of 138 clinical, questionnaire and experimental/technical measures within R2D2-MH cohorts were linked to the ICF. The full linking results for each measure are available in the published dataset.^25^ It is anticipated that shared codes across measures provide indications of where items may be readily harmonised. As a demonstrative example, several measures were linked to the ICF body functions code b1255 ‘Approachability’ (individual disposition to approach persons or things as opposed to withdrawing), which indicates that these items could potentially be harmonised. Within the main text of the manuscript, we only summarise the linking results. Here we divided measures into eight measurement categories based on their intended purpose to assist in summarising the linking results: (1) general intellectual abilities (k=9), (2) other cognitive and neuropsychological abilities and profiles (k=70), (3) medical and psychiatric symptoms (k=13), (4) measures of environmental factors (k=7), (5) background measures (k=6), (6) neurodevelopmental conditions or traits (k=24), (7) adaptive functioning (k=3) and (8) quality of life (k=6). The assigned category for each measure is shown in online supplemental table 2 and the total number of codes applied at the domain and chapter level across the eight measurement categories is displayed in online supplemental table 5. Figure 4a shows the distribution of codes across each of the eight measurement categories and their mapping onto the ICF domains. Most measures were linked to multiple domains of the ICF; however, measures of general intellectual ability and adaptive functioning showed a high distribution of codes in one domain only (body functions and activities and participation domains, respectively), with fewer codes across other domains. Body structures were not linked to any measure. Figure 4b similarly shows the distribution of codes to the ICF domains but presents domain mapping for each of the individual measures (represented by black dots in the figure). As anticipated, body functions and activity and participation domains were most frequently represented in the linking. Figure 4c shows the distribution of ICF codes for each of the eight measurement categories at a finer level of detail (third ICF level). The appearance of these shared codes across measures provides areas where measurement items might be readily harmonised. The most commonly occurring codes were perceptual functions (b156), linked to 38% of measures, and attention (b140) and basic interpersonal interaction (d710) functions, both linked to 32% of measures.

## Discussion

Here we propose using the ICF as a conceptual framework for the harmonisation of large-scale datasets in neurological diversity and present the first steps for how the ICF may be used to retrospectively harmonise diverse and multimodal datasets. Through the linking presented, we demonstrate how data collected under different paradigms may be aggregated under approaches that align with more contemporary approaches in psychiatry. For instance, we show that data embedded in more discrete diagnostic categories and psychopathology (eg, measures of clinical symptomology) can be transformed into functioning to facilitate more continuous and transdiagnostic approaches that are capable of examining challenges and strengths. For example, rather than focusing on the ‘severity’ of symptoms that an individual with a specific diagnosis may experience, the research could instead explore challenges in social communication and interpersonal interaction, or strengths in memory or attention which may also be present to varying degrees in other human populations.^5^ These approaches that break down diagnostic categories may be transformative for precision approaches in psychiatry by accounting for an individual’s unique needs, strengths, development and environmental contexts.^5^

Transforming symptomology and psychopathology-based data through a functioning framework may also support research outcomes to be more readily applicable to the day-to-day needs and challenges experienced by individuals and families.^12^ From a practical perspective, researchers can use the conceptual framework for harmonisation we present here to re-examine their existing datasets through a ‘functioning lens’, enabling exploration of new and unique research questions. Such investigations could lead to new insights that can inform clinical practice that promotes the functioning and well-being of neurologically diverse individuals. Stakeholder perspectives on research are also increasingly important for scientists to consider, and there are demands to ensure that research outputs are palatable to community stakeholders. From this standpoint, the shared language of the ICF and its compatibility with paradigms like neurodiversity may assist scientists to conduct research of relevance to stakeholders while simultaneously assisting in communicating and disseminating research and its findings. Although the process we present is primarily aimed at supporting the harmonisation of mental health data, the harmonisation results might also be beneficial for clinicians, where the measures they commonly use in clinical practice could be explored through a functioning lens to assist in extracting relevant information to support their clients.

It should be noted, however, that harmonising data will not eliminate all issues associated with more psychopathological framings of neurological diversity. The ICF seeks to prioritise primary perspectives (perspectives of the individual with neurological diversity), can capture both challenges and strengths and views the consideration of environmental factors (social, attitudinal, political and physical) as essential. Unfortunately, if original datasets do not capture these factors (eg, does not capture primary perspectives, strengths or environmental factors), they will not appear in harmonised datasets. Thus, though the ICF does present many advantages and opportunities, its potential is still dependent on the original data collected. This seems to be the case in our current harmonisation process, where few environmental codes were applied, likely reflecting the historical focus on biological and ‘psychopathological’ framings of the individual datasets. Resultantly, despite various advantages of retrospectively harmonising datasets using the ICF, researchers must remain cognisant that currently available mental health datasets may not enable full utilisation of the ICF’s potential. For this reason, new datasets, developed in collaboration with neurologically diverse individuals and capturing broader environmental influences, are also necessary.

Harmonising data according to the ICF may not be beneficial in all cases, and the ICF cannot cover all possible concepts or may not be specific enough in all cases. Other investigations may also contain information that is highly relevant to neurodevelopment and mental health but falls outside the scope of the ICF. For example, within the context of the datasets included in this harmonisation process, specific biological and genetic samples were taken for biomarker investigation. In this case, the ICF is unlikely to provide the level of detail necessary to provide a helpful basis for harmonisation. The diagnostic neutrality of the ICF, though representing a strength, also means that it does not operationalise psychopathology or mental health symptoms, which may also still be of interest to specific research questions. To retain this additional information which may remain relevant for investigation in neurodevelopment and mental health, other harmonisation methods for such data could be used to supplement ICF linking. Various procedures exist for these efforts and should be tailored to the specific research question and data, following the Maelstrom Research guidelines for rigorous retrospective data harmonisation.^26^ Other classification systems may also prove useful such as the International Classification of Diseases 11th Revision, which the ICF was designed to complement.^27^

Within the current manuscript, our main goal was to provide a comprehensive reflection on the use of the ICF as a conceptual framework for content harmonisation. This first step is essential to lay the basis for future data harmonisation efforts. There are several additional steps and challenges that must be addressed before the ICF can be suitably used for data harmonisation and generation of big data. The ICF provides a means to harmonise the content of measurements at an item level, enabling constructs across different measures and datasets to be standardised under a common framework. However, measurement invariance is necessary to prove that items are capturing similar constructs and thus can be reasonably harmonised. Here, structural equation modelling approaches may be applied. Quantitative harmonisation is also necessary to perform. Here we present several suggestions. First, variables could be converted, as far as practically possible, to a common rating scale. For example, the WHO suggests using qualifiers to rate the nature and magnitude of a problem,^13^ while other works have proposed the application of a numerical rating scale.^28^ Other methods that may be applied include standardising scores or equipercentile equating methods based on percentiles.^29^ These methods may however not be suitable or practical for many datasets. In these cases, more sophisticated methods are likely required. Regardless of the data processing method applied, some key issues must be considered which will be the focus of future work undertaken within the R2D2-MH consortium. The content harmonisation presented here is nevertheless anticipated to be helpful to researchers within the field to aid in their harmonisation efforts.

### Limitations

Although we believe that we have presented a compelling proposal that the ICF can facilitate harmonisation methods that align with contemporary approaches in psychiatry, several limitations must be considered. First, regarding content harmonisation, ensuring consistency and reliability in unifying variables is required. Our method presents some advantages here, but there remains potential for human error and variability when assigning ICF codes to items which may impact the consistency and reliability of the linking. At the same time, linking rules may not always be specific enough, and extracting concepts from diverse multimodal sources created to fulfil diverse purposes may also be challenging, as well as the differing perspectives in which information is collected. To address these limitations, resources and training on the application of the linking rules from the ICF Research Branch (https://www.icf-research-branch.org/icf-training/icf-workshops) should be used, and inter-rater linking could be performed on the full dataset. Future efforts that make use of machine learning may one day also assist in reducing subjectivity in the coding process. We are committed to full transparency and present all completed linking in ^25^ to enable researchers to both use the linking we present, and to enable researchers to relink for their own purposes in cases of disagreement.

## Conclusion

Ensuring harmonisation methods can align with contemporary paradigms in psychiatry is necessary to advance the field. Here, we propose that the ICF can provide a conceptual framework to support harmonising clinical and phenotypic data drawn from multiple and diverse datasets, enabling data to be re-examined through the biopsychosocial lens of functioning, which aligns with several contemporary paradigms in psychiatry.

### Clinical implications

Using the ICF as a conceptual framework for data harmonisation in psychiatry may provide a means of generating data that is compatible with contemporary approaches in psychiatry, being more capable of capturing diversity and aligning with more transdiagnostic and neurodiversity-affirmative ways of understanding data. Such approaches may assist in supporting future research that aims to support neurologically diverse individuals.

## Supplementary material

10.1136/bmjment-2024-301283 online supplemental file 1

## Data Availability

Data are available in a public, open access repository.
