# Supplementary material for: Conceptual framework for data harmonisation in mental health using the International Classification of Functioning, Disability and Health: an example with the R2D2-MH consortium
Source: BMJ Ment Health. 2024 Nov 28;27(1):e301283. doi: 10.1136/bmjment-2024-301283 (PMC11603809; doi:10.1136/bmjment-2024-301283)
Supplement: online supplemental file 1 [file bmjment-27-1-s001.pdf]

Supplementary Material

Data Sources

Within the context of the R2D2-MH, we used the ICF to harmonize data across cohorts. Work package leaders in R2D2-MH first compiled lists of candidate datasets, detailing the purposes, samples, availability of data, and variables of interest. Within this manuscript, we use an initial selection of a subset of datasets selected for inclusion in R2D2-MH. These datasets included the EU-AIMS Longitudinal European Autism Project (LEAP), [1] The Preschool Brain Imaging and Behaviour Project (PIP) https://www.aims-2-trials.eu/pip/, Evaluation of Magnetic Resonance Imaging to Predict Neurodevelopmental Impairment in Preterm Infants (ePRIME), [2] the SOSTA-net Randomized Controlled Trial (RCT), [3] the ASD-specific Frankfurt Early Intervention Programme for ASD RCT (A-FFIP), [4] the Neurobiology and Treatment of Adolescent Female Conduct Disorder (FemNat-CD), [5] and the developing Human Connectome Project (dHCP). [6] The datasets cover a broad range of cohorts in neurodevelopmental conditions and mental health, including diagnoses of autism, ADHD, intellectual disability, developmental delay, epilepsy, and conduct disorders, with ages ranging from infants born pre-term to adults. Most of the datasets also included neurotypical comparative samples. Additional measures planned to be conducted as part of R2D2-MH were also identified by the work package leaders for inclusion. A description of each study is provided in **Supplementary Table 1**, while a complete list of all measures is contained in **Supplementary Table 2.** In conducting the current harmonization process, only measures that contained information deemed to fall within the "ICF universe" (i.e., contained aspects relevant to functioning) were included. Measures and sources of information within datasets that did not contain information within the ICF universe (i.e., biological samples, medical or family histories) were not included.

Supplementary Table 1. Summary of studies

Study	Purpose	Populations	Country
ASD-specific Frankfurt Early Intervention Programme for ASD RCT (A-FFIP) [4]	Multi-center parallel-group, randomized controlled trial of the A-FFIP intervention.	Children aged 2 to 5.5 years who meet diagnostic criteria for autism (N=134).	Germany
AIMS-2 Trials Preschool Brain Imaging and Behaviour Project (PIP) (https://www.candy-project.eu/) (https://www.aims-2-trials.eu/pip/)	Investigate the mechanisms underlying the links between neurodevelopmental and co-occurring conditions and examine how treatments and monitoring can be improved by identifying biomarkers.	Children aged 2.5 years to 5.5 years who are neurotypical or have a diagnosis of autism, ADHD, or developmental delay.	England; Netherlands, Sweden, Belgium, France
Developing Human Connectome Project (dHCP) [6]	Map human brain connectivity in infants during pregnancy and after birth.	Pregnant woman with fetal age between 20-42 weeks gestational age and infants between 23- and 44-weeks gestational age (N~1000). Includes neurotypical and neurodivergent development (i.e., higher likelihood of neurodivergence to pre-term).	England
EU-AIMS Longitudinal European Autism Project (LEAP) [1, 7]	Identify factors that contribute to differences in brain development, social difficulties and autism characteristics and to identify stratification biomarkers.	Individuals aged 6 to 30 years who have a diagnosis of autism (n=437) or are neurotypical (n=300).	England, Netherlands, Germany, Sweden, Italy
Evaluation of Magnetic Resonance Imaging to Predict Neurodevelopmental Impairment in Preterm Infants (ePRIME) [2]	Examine how Magnetic Resonance Imaging may predict neurodevelopmental outcomes in infants born pre-term.	Infants born before 33 weeks gestation (N=511).	England
Neurobiology and Treatment of Adolescent Female Conduct Disorder (FemNat-CD) [5]	Examine sex differences in brain structure and function, hormones genetics, emotion recognition and regulation, and physiological activity, health and behavior, and examine impact of environment on well-being.	Children and adolescents with conduct disorder (n=855) and neurotypical (n=933) aged 9 to 18 years.	Germany, Switzerland, Netherlands
SOSTA-net RCT [3]	Multi-centre RCT evaluating ASD specific, group-based cognitive behavioural SOSTA-FRA approach	Autistic children and adolescents aged 8 – 19 years (N=209)	Germany

Supplementary Table 2. Measures included in linking procedure.

Measure	Category	Type	Rater		
			Caregiver	Self	Clinician
Bayley Scales III	A	C			X
Mullen Scales of Early Learning (MSEL)	A	C			X
Vineland Adaptive Behaviour Scale (VABS)	A	C			X
Brief Life Events Questionnaire (BLEQ)	B	Q	X		
Child Sleep Habits Questionnaire (CSHQ)	B	Q	X		
Childhood Intervention History (CIH)	B	Q	X		
COVID - 19 Health/Exposure Status	B	Q	X	X	
Edinburgh Handedness Inventory	B	Q	X		
Sleep Habits Questionnaire – Adults	B	Q		X	
Animated Shapes Narrative Task	CN	ET			X
Animated Shapes Narrative Task with fMRI	CN	ET			X
Auditory Amplitude Discrimination (Songbirds)	CN	ET			X
Auditory Frequency Discrimination (Songbirds)	CN	ET			X
Behavior Rating Inventory of Executive Function-Preschool version (BRIEF-P)	CN	Q	X		
Biological Motion	CN	ET			X
CANTAB Intra-Extra Dimensional Set Shift	CN	ET			X
CANTAB One Touch Stockings of Cambridge	CN	ET			X
CANTAB Paired Associates Learning (PAL)	CN	ET			X
CANTAB Rapid Visual Information Processing (RVP)	CN	ET			X
CANTAB Spatial Span	CN	ET			X
CANTAB Spatial Working Memory	CN	ET			X
Change Detection - Spot the difference	CN	ET			X
Child Behaviour Checklist (CBCL) 1.5 - 5 years	CN	Q		X	
Child Behaviour Checklist (CBCL) 6 - 18 years	CN	Q	X		
Child Empathising - Systemizing (EQ-SQ)	CN	Q	X		
Child Youth and Resilience Measure (CYRM)	CN	Q	X		
Childhood Behaviour Questionnaire (CBQ)	CN	Q	X		
Columbia Impairment Scale (CIS)	CN	Q	X	X	
Delay of Gratification	CN	ET			X
Early Childhood Behavior Questionnaire (ECBQ) – Short	CN	Q	X		
Emotion Dot probe (Face n the crowd)	CN	ET			X
Emotion Matching	CN	ET			X
Emotion Recognition Task (Good days, bad days)	CN	ET			X
Emotion Regulation Checklist	CN	Q	X		
Empathy Quotient (EQ)	CN	Q		X	
Event Memory	CN	ET			X
False belief (Pips bus)	CN	ET			X
False Belief (Whats a modi?)	CN	ET			X
Film Expression	CN	ET			X
Flanker Go/No-Go task	CN	ET			X
fMRI Social Non-Social Reward	CN	ET			X
Gap Overlap	CN	ET			X
Glitter wand	CN	ET			X

Measure	Category	Type		Rater	
Go-No-Go (Find the puppy)	CN	ET			X
Hariri emotion processing with fMRI	CN	ET			X
High Sensitivity Child Scale (HSCS)	CN	Q	X		
Highly Sensitive Personal Scale (HSPS)	CN	Q	X	X	
Implicit False Belief Task	CN	ET			X
Implicit Learning (Hopping frog)	CN	ET			X
Inferring Desire (What does Pip like?	CN	ET			X
Intolerance of Uncertainty Questionnaire	CN	Q	X		
Irritability Questionnaire	CN	Q	X		
Karolinska Directed Emotional Faces (KDEF)	CN	ET			X
Motion Coherence (Flying Spaceships)	CN	ET			X
Natural scenes: static and dynamic	CN	ET			X
Probabilistic Reversal Learning	CN	ET			X
Pupillary Light Reflex	CN	ET			X
Reactive-Proactive Aggression Questionnaire	CN	Q		X	
Reading the Mind in the Eyes Task	CN	ET			X
Reinforcement Learning (Magic Boxes)	CN	ET			X
Sandbox continuous false belief task	CN	ET			X
Sensory Experiences Questionnaire (SEQ)	CN	Q	X		
Short Sensory Profile (SSP)	CN	Q	X		
Simple Reaction Time task (Bubble pop)	CN	ET			X
Social Non Social Videos	CN	ET			X
Social Reinforcement Learning (Come play with us)	CN	ET			X
Spatial Working Memory	CN	ET			X
Strengths and Difficulties Questionnaire (SDQ) - 11 - 17 years	CN	Q	X	X	
Strengths and Difficulties Questionnaire (SDQ)- 4 - 10 years	CN	Q	X		
Strengths and Difficulties Questionnaire (SDQ)- 2 - 4 years	CN	Q	X		
Sustained Attention (Pips car)	CN	ET		X	
Systemizing Quotient (SQ)	CN	Q	X		X
Tactile Discrimination task (sleepy kitties)	CN	ET			X
Tactile Gating (EEG)	CN	ET			X
Temporal Discounting	CN	ET			X
Toronto Alexithymia Scale	CN	Q	X		X
Un/Segmented Block Design	CN	ET			X
Upright Inverted faces N170	CN	ET			X
Upright/Inverted faces Gamma	CN	ET			X
Bullying and Friendship Interview (BFIS)	E	C			X
Cognitively Stimulating Parenting Scale (CSPS)	E	Q	X		
HOME Scale - Under 3	E	Q	X		
HOME Scale 10 - 14 years	E	Q	X		
HOME Scale 3-5 years	E	Q	X		
HOME Scale 6 - 9 years	E	Q	X		
Multidimensional Scale of Perceived Social Support (MSPSS)	E	Q		X	
British Picture Vocabulary Scale (BPVS)	IA	C			X
Hansen Research Services Matrix Adaptive Test (HRS-MAT)	IA	C		X	

Measure	Category	Type	Rater		
Parent report of Childrens Abilities - Revised (PARCA-R)	IA	Q	X		
Ravens Coloured Progressive Matrices	IA	C			X
The Wechsler Intelligence Scale for Children - Fourth Edition (WISC-IV)	IA	C			X
Wechsler Abbreviated Intelligence Scale (WASI)	IA	C			X
Wechsler Adult Intelligence Scale - Fourth Edition (WAIS-IV)	IA	C			X
Wechsler Preschool and Primary Scale of Intelligence (WPPSI) - 2 - 3 years	IA	C			X
Wechsler Preschool and Primary Scale of Intelligence (WPPSI) - 4 - 7 years	IA	C			X
Aberrant Behaviour Checklist (ABC)	MP	Q	X		
Beck Anxiety Inventory (BAI)	MP	Q		X	
Beck Depression Inventory (BDI)	MP	Q		X	
Child Health and Illness Profile (CHIP-CE)	MP	Q		X	
Depression Anxiety and Stress Scale (DASS - 21)	MP	Q		X	
Development and Well-being Assessment (DAWBA) - Parent report	MP	C	X	X	X
Development and Well-being Assessment (DAWBA) – Self-report	MP	Q	X		
General Health Questionnaire	MP	Q	X		
Gross Motor Function Classification System	MP	C			X
Hamilton Depression Rating Scale (HDRS)	MP	Q			X
Kiddie Schedule for Affective Disorders and Schizophrenia (K-SADS)	MP	C			X
Oxford-Liverpool Inventory of Feelings and Experiences (O-LIFE)	MP	Q		X	
State -Trait Anxiety Inventory (STAI)	MP	Q			X
Adult Routines Inventory (ARI)	NDC	Q		X	
Adults' Social Behaviour Questionnaire (ASBQ)	NDC	Q	X	X	
Autism Diagnostic Interview - Revised (ADI-R)	NDC	C			X
Autism Diagnostic Observation Schedule (ADOS) Module 1	NDC	C			X
Autism Diagnostic Observation Schedule (ADOS) Module 2	NDC	C			X
Autism Diagnostic Observation Schedule (ADOS) Module 3	NDC	C			X
Autism Diagnostic Observation Schedule (ADOS) Module 4	NDC	C			X
Autism Quotient (AQ)	NDC	Q		X	
Autism Quotient (AQ) - Adolescent	NDC	Q	X		
Autism Quotient (AQ) - Child	NDC	Q	X		
Brief Observation of Social Communication Change (BOSCC)	NDC	C			X
Childhood Routines Inventory - Revised (CRI-R)	NDC	Q	X		
Childrens Social Behaviour Questionnaire (CSBQ)	NDC	Q			X
Developmental Coordination Questionnaire (DCDQ)	NDC	Q	X		

Measure	Category	Type	Rater		
DSM-5 ADHD Rating Scale	NDC	Q	X		
Dyadic Communication Measure for Autism	NDC	C			X
Early Social Communication Scale	NDC	C			X
Modified Autism Checklist (M-CHAT)	NDC	Q	X		
Quantitative Checklist for Autism in Toddlers (Q-CHAT)	NDC	Q	X		
Repetitive Behaviour Scale	NDC	Q	X		
Social Communication Questionnaire (SCQ)	NDC	Q	X		
Social Responsiveness Scale (SRS)	NDC	Q	X		
Social Responsiveness Scale (SRS) - Preschool	NDC	Q	X		
Social Responsiveness Scale (SRS) - Short form	NDC	Q		X	
EQ-5D-3L	Q	Q	X		
Family Quality of Life Survey (FQOLS)	Q	Q	X		
KIDDYKINDL - Parent report	Q	Q	X		
Kidscreen - 10	Q	Q		X	
SF-12	Q	Q		X	
WHO-QOL BREF	Q	Q			X

Note. Category refers to the category that measures were allocated based on their intended purpose. Categories include Adaptive functioning (A), Background measures (B), Other cognitive and neuropsychological abilities and profiles (CN), Environmental factors (E), General intellectual abilities (IA), Medical and psychiatric symptoms (MP), neurodevelopmental conditions or traits (NDC), and Quality of life measures (Q). Type refers to the type of measurement. Measurement types include clinical measures (C) such as those performed by clinicians for the purposes of evaluating behaviors or diagnosing conditions. Questionnaires (Q) are rating scales or forms completed by individuals, their caregivers or clinicians. Experimental or technical measures (ET) are those measures that are typically completed in a laboratory-based setting to measure performance on various tasks. Rater refers to the individual administering (in the case of clinical or experimental/technical measures) or scoring the measure (for questionnaires).

Linking procedure

Linking of measures to the ICF was conducted in accordance with ICF linking guidelines established by the WHO and ICF research branch. [8, 9] For questionnaires, identified measures were located, and individual items were extracted. [8] Each item was then reviewed within the broader context and purpose of the measure to identify the concept most relevant to be linked (main concept), as well as additional concepts containing other relevant information. For performance measures (i.e., cognitive tasks), the aim of the measurement was identified as the main concept. [9] As recommended by the most recent linking guidelines, the perspectives and response options were also extracted from the measures. Perspectives refer to the underlying purpose of the measure and were categorized according to whether the measure sought to capture a descriptive, appraisal or need/dependency perspective according to definitions provided by Cieza et al [8] Measures examining descriptive perspectives could refer to performance or capacity, where performance captures an individual's *actual* performance in their environment (which can be supporting or hindering performance) and

capacity captures an individual's *ability* to complete tasks without the impact of the environment (supposed to capture an individual's highest level of performance). Measures using appraisal perspectives explore the degree to which expectations are met, while need/dependency perspectives capture the degree and type of dependencies of an individual (i.e., support needs). [8] Response options were also extracted and categorized as either intensity, frequency, duration, confirmation/agreement, or qualitative attributes. [8]

The extracted main and additional concepts were subsequently linked to the ICF by applying established ICF linking rules and decision-making processes [8] Here, we used the ICF – Child and Youth version (-CY) as this version represents the most comprehensive version of the ICF, containing codes also relevant to both adults and developing individuals. [10] First, the extracted main and additional concepts were examined to determine whether they belonged within the ICF universe and could be assigned to an ICF component. Where concepts were deemed not to belong to the ICF universe, they were coded as "not codable." Where concepts belonged to the ICF universe, they were coded according to the most precise ICF code, with "other specified" and "unspecified codes" used as necessary. Concepts that did not provide sufficient information to determine the most appropriate ICF code were coded as "not definable". Personal factors (i.e., age, sex/gender, cultural background) are ordinarily coded as "personal factor" according to ICF linking rules, but to capture this information, concepts identified as personal factors were linked to the personal factor classification system developed by Grotkamp et al. [11] An overview of the linking process is displayed in **Supplementary Figure 1**. Examples of the main and additional concepts extracted from the measures, as well as their corresponding ICF codes, are shown in **Supplementary Tables 3 and 4**. Given that datasets drawn on for harmonization purposes may not always contain item-level data. Scale-level data was also extracted and linked to the ICF following the same process described for item-level data. Scale-level linking is not presented in this manuscript but is contained within the supplementary materials.

The linking process was completed by a researcher who had received training from the WHO research branch on ICF linking and who has expertise in ICF linking methodology in consultation with members of the research team and another researcher with extensive expertise in the ICF (SB). To improve the reliability of the linking process, ICF linking for a subset of items (1599 items. From 22 measures) was compared to linking performed independently by two secondary linkers, also experienced with ICF methodology. Codes assigned to each of the 1599 items were compared at the second-level and then assigned a binary classification of 1 (yes, agreement) or 0 (no agreement). Following the calculation of the inter-rater agreement, areas of discrepancy were observed and resolved, in consultation with

an additional reviewer (SB) if needed. Linking for the remaining measures and items were then refined, corrected, and finalized based on the results of the inter-rater linking. When calculated at the second ICF level, inter-rater agreement for these measures was high (75%), with Cohens Kappa indicating substantial agreement (k=0.75, CIs: k= 0.73 – 0.77), suggesting that the linking was reliable. To enhance transparency and to enable researchers to utilize the linking, we provide all linking at the item and scale level in the supplement. For the purposes of results presentation, linking is reported as absolute and relative frequencies at the domain, chapter, and second level of the ICF. Visualizations of the linking at a domain level were also developed using Gephi [12] and the sigma.js plugin for Gephi developed by the InteractiveVis project of the Oxford Internet Institute. [13]

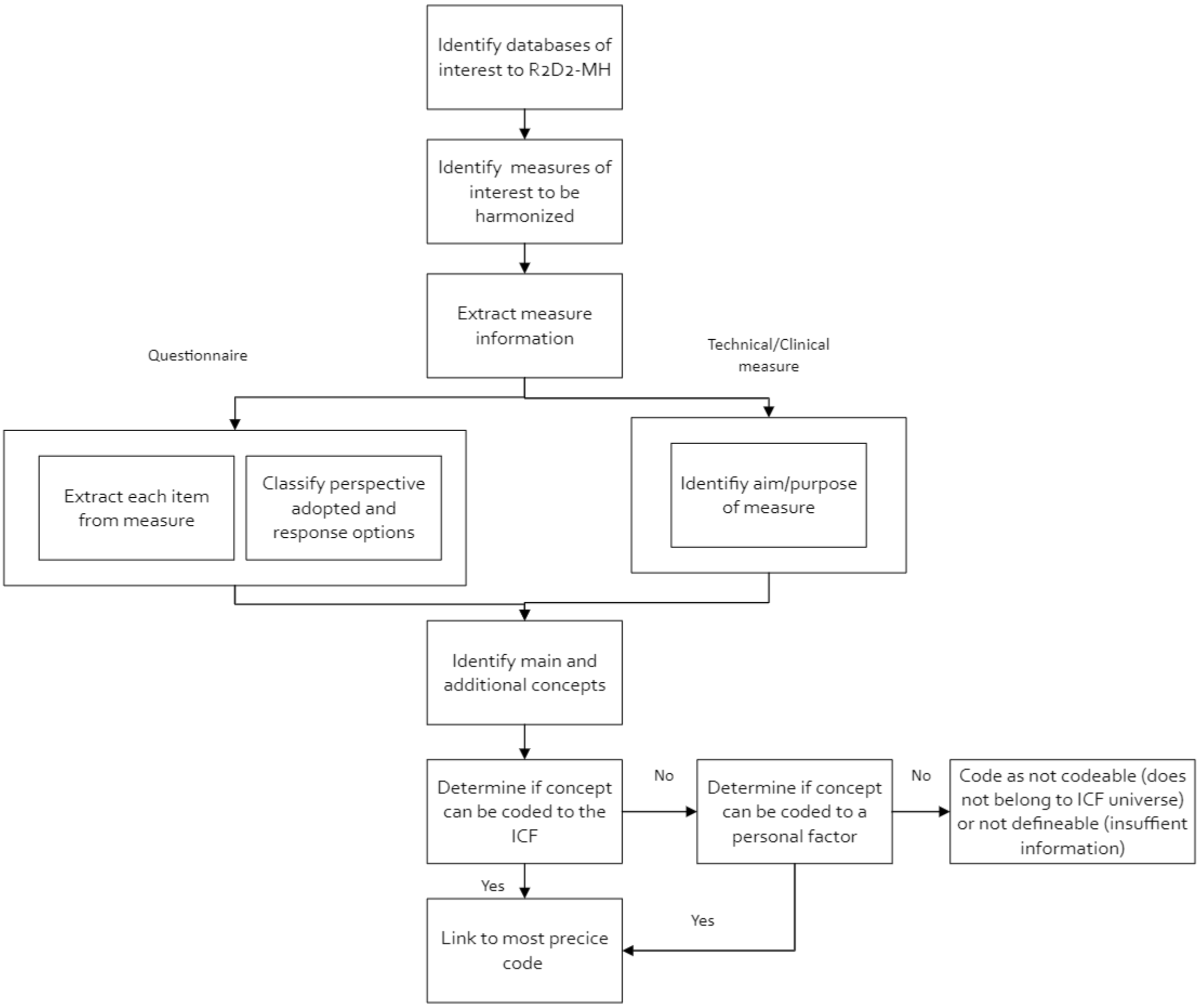

Supplementary Figure 1. Overview of concept harmonization process based on [9].

Supplementary Table 3. Example of linking extraction for questionnaire measures

Item	Scale	Perspective	Response option	Response option classification	Main concepts and *additional concepts*	ICF Code for Main concepts and *additional concepts*
"Pursues movement to the point it interferes with daily routines (for example, can't sit still, fidgets)."	Short Sensory Profile	Descriptive - performance	Does not apply/Almost never/Occasionally/Half the time/Frequently/Almost always	Frequency	Pursuit of movement *Interference with daily routines*	b1470 – Psychomotor control *d2308 - Carrying out daily routine, other specified*
"Good attention span sees work through to the end."	Strengths and Difficulties Questionnaire	Descriptive - performance	Not true/Somewhat true/Certainly true	Agreement	Attention span *Completing tasks*	b1400 - Sustaining attention *d2105 – completing complex tasks*
Pointing (socially-directed pointing)	Autism Diagnostic Observation Schedule	Descriptive – capacity	0-3 rating, where "0" is the child uses socially directed pointing, and "3" is the child not does not point as described.	Intensity	Pointing for communication	d3350 -Producing body language
Gender	Kiddie Schedule for Affective Disorders and Schizophrenia	Descriptive	Male/Female	Qualitative attribute	Biological sex (male, female)	i120 - Biological sex

Supplementary Table 4. Example of linking extraction for technical and clinical measures.

**Technical/Clinical measure**	**Aim/Purpose**	**Main concept and *additional concepts***	**ICF Code for Main Concept and *additional concepts***
Karolinska Directed Emotional Faces (KDEF)	Measure facial emotion recognition abilities	Facial Emotion Recognition	b16703 - Reception of body language
Sandbox continuous false belief task	Examine theory of mind/mentalizing	Theory of mind	b122 Global Psychosocial functions
CANTAB Rapid Visual Information Processing (RVP)	Measure sustained attention	Sustained attention	b1400 - Sustaining attention

Results

A total of 138 clinical, questionnaire, and experimental/technical measures within R2D2-MH cohorts were linked to the ICF. Here we divided measures into eight measurement categories based on their intended purpose to assist in summarizing the linking results. 1, General intellectual abilities (k=9; e.g., Wechsler Abbreviated Intelligence Scale[14]), 2, Other cognitive and neuropsychological abilities and profiles (k=70; e.g., Reading the Mind in the Eyes Test[15]), 3, Medical and psychiatric symptoms (k=13; e.g., Beck Depression Inventory[16]), 4, Measures of environmental factors (k=7; e.g., Cognitively Stimulating Parenting Scale[17]), 5, Background measures (k=6; e.g., Brief Life Events Questionnaire[18]), 6, Neurodevelopmental conditions or traits (k=24; e.g., Autism Diagnostic Interview – Revised[19]), 7, Adaptive functioning (k=3; e.g., Vineland Adaptive Behaviour Scales[20]) and 8, Quality of life (k=6; e.g., WHO-QoL[21]). **Supplementary Table 5** presents the number of codes applied at the domain and chapter level across the eight measurement categories.

Supplementary Table 5. Number of codes applied at the domain and chapter level across the eight measurement categories.

	Background	Cognitive and Psychological Profile	Environment	General intellectual ability and language	Medical/Psychiatric	Neurodevelopmental condition diagnosis/traits	Quality of life	Adaptive Functioning
**Body Functions**	119	576	10	182	1184	637	47	136
b1 Mental functions	107	533	10	181	958	563	42	112
b2 Sensory functions and pain	4	15	0	0	22	10	5	5
b3 Voice and speech functions	0	2	0	0	7	14	0	5
b4 Functions of the cardiovascular, haematological, immunological and respiratory systems	5	4	0	0	22	2	0	0
b5 Functions of digestive, metabolic and endocrine systems	0	8	0	0	65	3	0	3
b6 Genitourinary and reproductive functions	0	5	0	1	20	0	0	0
b7 Neuromusculoskeletal and movement-related functions	2	7	0	0	82	45	0	11
b8 Functions of the skin and related structures	1	2	0	0	8	0	0	0
**Activities and Participation**	36	268	253	35	617	554	120	591
d1 Learning and applying knowledge	2	39	26	13	35	93	0	112
d2 General tasks and demands	9	89	0	0	96	81	8	23
d3 Communication	1	8	13	11	26	129	0	123
d4 Mobility	10	2	0	8	92	11	7	160
d5 Self-care	2	22	6	0	115	10	4	55
d6 Domestic life	0	0	11	0	1	2	23	27
d7 Interpersonal interactions and relationships	5	77	182	0	150	189	32	56
d8 Major life areas	5	19	0	3	60	34	24	22
d9 Community, social and civic life	2	12	15	0	42	5	22	13

	Background	Cognitive and Psychological Profile	Environment	General intellectual ability and language	Medical/Psychiatric	Neurodevelopmental condition diagnosis/traits	Quality of life	Adaptive Functioning
**Environmental Factors**	91	24	215	0	173	12	127	0
e1 Products and technology	14	1	19	0	42	0	14	0
e2 Natural environment and human-made changes to environment	0	3	3	0	0	0	0	0
e3 Support and relationships	10	18	189	0	105	9	59	0
e4 Attitudes	0	1	0	0	14	3	3	0
e5 Services, systems and policies	67	1	4	0	12	0	51	0
**Personal Factors**	16	10	1	0	96	26	0	0
i1 General personal characteristics	0	0	0	0	1	0	0	0
i2 Physical factors	0	0	0	0	16	0	0	0
i3 Mental factors	0	0	0	0	0	0	0	0
i4 Attitudes, action-related skills, and behavior patterns	12	10	1	0	71	26	0	0
i5 Life situation	4	0	0	0	8	0	0	0

References

1. Loth E, Charman T, Mason L, Tillmann J, Jones EJH, Wooldridge C, Ahmad J, Auyeung B, Brogna C, Ambrosino S, Banaschewski T, Baron-Cohen S, Baumeister S, Beckmann C, Brammer M, Brandeis D, Bölte S, Bourgeron T, Bours C, de Bruijn Y, Chakrabarti B, Crawley D, Cornelissen I, Acqua FD, Dumas G, Durston S, Ecker C, Faulkner J, Frouin V, Garces P, Goyard D, Hayward H, Ham LM, Hipp J, Holt RJ, Johnson MH, Isaksson J, Kundu P, Lai M-C, D'ardhuy XL, Lombardo MV, Lythgoe DJ, Mandl R, Meyer-Lindenberg A, Moessnang C, Mueller N, O'Dwyer L, Oldehinkel M, Oranje B, Pandina G, Persico AM, Ruigrok ANV, Ruggeri B, Sabet J, Sacco R, Cáceres ASJ, Simonoff E, Toro R, Tost H, Waldman J, Williams SCR, Zwiers MP, Spooren W, Murphy DGM, Buitelaar JK. The EU-AIMS Longitudinal European Autism Project (LEAP): design and methodologies to identify and validate stratification biomarkers for autism spectrum disorders. Mol Autism. 2017;8(1):24.
2. Edwards AD, Redshaw ME, Kennea N, Rivero-Arias O, Gonzales-Cinca N, Nongena P, Ederies M, Falconer S, Chew A, Omar O, Hardy P, Harvey ME, Eddama O, Hayward N, Wurie J, Azzopardi D, Rutherford MA, Counsell S, ePrime I. Effect of MRI on preterm infants and their families: a randomised trial with nested diagnostic and economic evaluation. Arch Dis Child Fetal Neonatal Ed. 2018;103(1):F15-F21.
3. Freitag CM, Jensen K, Elsuni L, Sachse M, Herpertz-Dahlmann B, Schulte-Rüther M, Hänig S, von Gontard A, Poustka L, Schad-Hansjosten T, Wenzl C, Sinzig J, Taurines R, Geißler J, Kieser M, Cholemkery H. Group-based cognitive behavioural psychotherapy for children and adolescents with ASD: the randomized, multicentre, controlled SOSTA-net trial. J Child Psychol Psychiatr. 2016;57(5):596-605.
4. Kitzerow J, Hackbusch M, Jensen K, Kieser M, Noterdaeme M, Fröhlich U, Taurines R, Geißler J, Wolff N, Roessner V, Bast N, Teufel K, Kim Z, Freitag CM. Study protocol of the multi-centre, randomised controlled trial of the Frankfurt Early Intervention Programme A-FFIP versus early intervention as usual for toddlers and preschool children with Autism Spectrum Disorder (A-FFIP study). Trials. 2020;21(1):217.
5. Freitag C. Neurobiology and treatment of adolescent female conduct disorder: FemNAT-CD consortium: a new European cooperation. Eur Child Adolesc Psychiatry. 2014;23(8):723-4.
6. Makropoulos A, Robinson EC, Schuh A, Wright R, Fitzgibbon S, Bozek J, Counsell SJ, Steinweg J, Vecchiato K, Passerat-Palmbach J, Lenz G, Mortari F, Tenev T, Duff EP, Bastiani M, Cordero-Grande L, Hughes E, Tusor N, Tournier J-D, Hutter J, Price AN, Teixeira RPAG, Murgasova M, Victor S, Kelly C, Rutherford MA, Smith SM, Edwards AD, Hajnal JV, Jenkinson M, Rueckert D. The developing human connectome project: A minimal processing pipeline for neonatal cortical surface reconstruction. NeuroImage. 2018;173:88-112.
7. Charman T, Loth E, Tillmann J, Crawley D, Wooldridge C, Goyard D, Ahmad J, Auyeung B, Ambrosino S, Banaschewski T, Baron-Cohen S, Baumeister S, Beckmann C, Bölte S, Bourgeron T, Bours C, Brammer M, Brandeis D, Brogna C, de Bruijn Y, Chakrabarti B, Cornelissen I, Acqua FD, Dumas G, Durston S, Ecker C, Faulkner J, Frouin V, Garcés P, Ham L, Hayward H, Hipp J, Holt RJ, Isaksson J, Johnson MH, Jones EJH, Kundu P, Lai M-C, D'ardhuy XL, Lombardo MV, Lythgoe DJ, Mandl R, Mason L, Meyer-Lindenberg A, Moessnang C, Mueller N, O'Dwyer L, Oldehinkel M, Oranje B, Pandina G, Persico AM, Ruggeri B, Ruigrok ANV, Sabet J, Sacco R, Cáceres ASJ, Simonoff E, Toro R, Tost H, Waldman J, Williams SCR, Zwiers MP, Spooren W, Murphy DGM, Buitelaar JK. The EU-AIMS Longitudinal European Autism Project (LEAP): clinical characterisation. Mol Autism. 2017;8(1):27.
8. Cieza A, Fayed N, Bickenbach J, Prodinger B. Refinements of the ICF linking rules to strengthen their potential for establishing comparability of health information. Disabil Rehabil. 2019;41(5):574-83.
9. Cieza A, Geyh S, Chatterji S, Kostanjsek N, Ustün B, Stucki G. ICF linking rules: An update based on lessons learned. J Rehabil Med. 2005;37(4):212-8.

10. WHO. International Classification of Functioning, Disability and Health: Children and Youth version: ICF-CY: World Health Organization; 2007.
11. Grotkamp S, Cibis W, Brüggemann S, Coenen M, Gmünder HP, Keller K, Nüchtern E, Schwegler U, Seger W, Staubli S, von Raison B, Weißmann R, Bahemann A, Fuchs H, Rink M, Schian M, Schmitt K. Personal factors classification revisited: A proposal in the light of the biopsychosocial model of the World Health Organization (WHO). Aust J Rehabil Couns. 2020;26(2):73-91.
12. Bastian M, Heymann S, Jacomy M, editors. Gephi: an open source software for exploring and manipulating networks. International AAAI Conference on Weblogs and Social Media; 2019.
13. Scott H. InteractiveVis: Updated plugin and future development. 2016.
14. Wechsler D. Wechsler Abbreviated Scale of Intelligence. San Antonio, TX: The Psychological Corporation; 1999.
15. Baron-Cohen S, Wheelwright S, Hill J, Raste Y, Plumb I. The "Reading the Mind in the Eyes" Test revised version: a study with normal adults, and adults with Asperger syndrome or high-functioning autism. J Child Psychol Psychiatr. 2001;42(2):241-51.
16. Beck AT, Ward CH, Mendelson M, Mock J, Erbaugh J. An inventory for measuring depression. Arch Gen Psychiatry. 1961;4:561-71.
17. Wolke D, Jaekel J, Hall J, Baumann N. Effects of sensitive parenting on the academic resilience of very preterm and very low birth weight adolescents. J Adolesc Health. 2013;53(5):642-7.
18. Brugha TS, Cragg D. The List of Threatening Experiences: the reliability and validity of a brief life events questionnaire. Acta Psychiatr Scand. 1990;82(1):77-81.
19. Rutter M, Le Couteur A, Lord C. The Autism Diagnostic Interview – Revised (ADI-R). Los Angeles: Western Psychological Service; 2003.
20. Sparrow SS, Cicchetti DV, Saulnier CA. Vineland Adaptive Behavior Scales: Third Edition (Vineland-3). Bloomington: NCS Pearson; 2016.
21. World Health Organization. The World Health Organization Quality of Life (WHOQOL) - BREF, 2012 revision. https://apps.who.int/iris/handle/10665/77773: World Health Organization; 2004.
